# Multi‐family therapy for eating disorders: A systematic scoping review of the quantitative and qualitative findings

**DOI:** 10.1002/eat.23616

**Published:** 2021-10-20

**Authors:** Julian Baudinet, Ivan Eisler, Lisa Dawson, Mima Simic, Ulrike Schmidt

**Affiliations:** ^1^ Institute of Psychiatry, Psychology and Neuroscience (IoPPN) King's College London London UK; ^2^ Maudsley Centre for Child and Adolescent Eating Disorders (MCCAED) South London and Maudsley NHS Foundation Trust London UK; ^3^ Eating Disorder Service Westmead Children's Hospital, Sydney Children's Hospital Network Sydney New South Wales Australia; ^4^ Adult Eating Disorders Service South London and Maudsley NHS Foundation Trust, De Crespigny Park Denmark Hill, London UK

**Keywords:** adolescent, adult, anorexia nervosa, bulimia nervosa, caregiver, child, eating disorders, family‐based treatment (FBT), Maudsley family therapy, multi‐family therapy (MFT), young adult

## Abstract

**Objective:**

This study reviewed the quantitative and qualitative evidence‐base for multi‐family therapy (MFT) for eating disorders regarding change in physical and psychological symptoms, broader individual and family factors, and the experience of treatment.

**Method:**

A systematic scoping review was conducted. Four databases (PsycInfo, Medline, Embase, CENTRAL) and five grey literature databases were searched on 24th June 2021 for relevant peer‐reviewed journal articles, book chapters, and dissertations. No beginning time‐point was specified. Only papers that presented quantitative or qualitative data were included. No restrictions on age or diagnosis were imposed. Studies were first mapped by study design, participant age, and treatment setting, then narratively synthesized.

**Results:**

Outcomes for 714 people who received MFT across 27 studies (one mixed‐method, 17 quantitative and nine qualitative) were synthesized. MFT is associated with improvements in eating disorder symptomatology and weight gain for those who are underweight. It is also associated with improvements in other individual and family factors including comorbidities, self‐esteem, quality of life, and some aspects of the experience of caregiving, although these findings are more mixed. MFT is generally experienced as both helpful and challenging due to the content addressed and intensive group process.

**Discussion:**

MFT is associated with significant improvements in eating disorder symptoms across the lifespan and improvement in broader individual and family factors. The evidence base is small and studies are generally underpowered. Larger, higher‐quality studies are needed, as is research investigating the unique contribution of MFT on outcomes, given it is typically an adjunctive treatment.

## Resumen

**Objetivo:**

Este estudio revisó la evidencia cuantitativa y cualitativa para la terapia multifamiliar (MFT, por sus siglas en inglés) para los trastornos de la conducta alimentaria con respecto al cambio en los síntomas físicos y psicológicos, los factores individuales y familiares más extensos, y la experiencia del tratamiento.

**Método:**

Se realizó una revisión sistemática del alcance. Se realizaron búsquedas en cuatro bases de datos (PsycInfo, Medline, Embase, CENTRAL) y en cinco bases de datos de literatura gris el 24.06.2021 para obtener artículos relevantes de revistas revisadas por pares, capítulos de libros y disertaciones. No se especificó ningún punto de tiempo inicial. Sólo se incluyeron los artículos que presentaban datos cuantitativos o cualitativos. No se impusieron restricciones de edad o diagnóstico. Los estudios se mapearon primero por el diseño del estudio, la edad de los participantes y el entorno de tratamiento, y luego se sintetizaron narrativamente.

**Resultados:**

Se sintetizaron los resultados de 714 pacientes que recibieron MFT en 27 estudios (un método mixto, 17 cuantitativos y nueve cualitativos). MFT se asocia con mejoras en la sintomatología del trastorno de la conducta alimentaria y el aumento de peso para aquellos que tienen bajo peso. También se asocia con mejoras en otros factores individuales y familiares, incluidas las comorbilidades, la autoestima, la calidad de vida y algunos aspectos de la experiencia del cuidador, aunque estos hallazgos son más mixtos. MFT generalmente se experimenta como útil y desafiante debido al contenido abordado y al proceso grupal intensivo.

**Discusión:**

La MFT se asocia con mejoras significativas en los síntomas del trastorno de la conducta alimentaria a lo largo de la vida y una mejora en factores individuales y familiares más amplios. La base de evidencia es pequeña y los estudios generalmente tienen poco poder. Se necesitan estudios más grandes y de mayor calidad, al igual que la investigación que investiga la contribución única de la MFT en los resultados, dado que generalmente es un tratamiento complementario.

**Palabras clave:**

terapia multifamiliar (MFT), terapia familiar de Maudsley, tratamiento basado en la familia (FBT), trastornos de la conducta alimentaria, anorexia nerviosa, bulimia nerviosa, niño, adolescente, adulto joven, adulto, cuidador

## 1 INTRODUCTION

Bringing families together to form multi‐family groups has been part of eating disorder treatment for decades (Gelin, Cook‐Darzens, & Hendrick, 2018). This emerged in the context of a longer tradition of multi‐family groups for people with schizophrenia (Laqueur, Laburt, & Morong, 1964; McFarlane, 2002), and more recent models for depression (Anderson et al., 1986; Lemmens, Eisler, Buysse, Heene, & Demyttenaere, 2009) and substance misuse (Kaufman & Kaufmann, 1977).

Early eating disorder multi‐family group work in the 1980s focused on young adults with anorexia nervosa (Slagerman & Yager, 1989) and bulimia nervosa (Wooley & Lewis, 1987). These early eating disorders focused multi‐family groups primarily targeted family relationships and improving patient support. In the 1990s, multi‐family group therapy (MFT) models began emerging for children and adolescents with eating disorders (Dare & Eisler, 2000; Scholz & Asen, 2001), which were theoretically rooted in the principles of eating disorder focused family therapy (Eisler, Simic, Blessitt, Dodge, & MCCAED Team, 2016).

This umbrella term, eating disorder‐focused family therapy, encompasses several, similar forms of evidence‐based family therapy for eating disorders, including Maudsley family therapy (Eisler et al., 2016) and family‐based treatment (Lock & Le Grange, 2012). While some differences exist, all eating disorder focused family therapies are phased, emphasize working with the family rather than *treating* the family, initially focus on symptom management with parents taking a central role supporting their child's eating, and broadens out to adolescent and family functioning once healthier food and eating practices are established and physical health has improved.

Different versions of MFT for anorexia nervosa and atypical anorexia nervosa (MFT‐AN) have now been manualized for children and adolescents (Simic, Baudinet, Blessitt, Wallis, & Eisler, 2021), as well as adults (Tantillo, McGraw, & Le Grange, 2020). Typically, MFT‐AN involves a group of up to eight families working together with at least two healthcare professionals. The group usually engages in a mixture of different types of activities, including small and large group discussions, nonverbal activities, and therapeutic games. This all occurs in various constellation, such as separate young person, sibling and parent groups, mixed groups, or pairs, etc. The group will usually also eat up to three meals together during each MFT day. MFT‐AN is now a recommended treatment for adolescents by several practice guidelines (Couturier et al., 2020; Heruc et al., 2020; NICE, 2017) and a specific version has recently been developed for adolescents with bulimia nervosa (MFT‐BN; Stewart et al., 2019).

Individual differences exist between MFT models (Gelin et al., 2018; Gelin, Cook‐Darzens, Simon, & Hendrick, 2016). Some offer three (Whitney et al., 2012) or five days (Knatz et al., 2015; Marzola et al., 2015; Wierenga et al., 2018) of MFT groups as a stand‐alone intervention. Others are much longer and offer 10 (Simic et al., 2021) or even 20 days or more (Gelin et al., 2016; Scholz, Rix, Scholz, Gantchev, & Thömke, 2005) of MFT groups spread across 12 months with reducing frequency. The number of families per group also varies, ranging from two (Whitney, Murphy, et al., 2012) to eight or nine (Simic et al., 2021). Similarly, MFT intensity is variable, with some meeting weekly/fortnightly for 1–2 hours (Gelin et al., 2016; Stewart et al., 2019) and others for several full consecutive days (Scholz et al., 2005).

All MFT models are designed to improve treatment outcomes by reducing perceived isolation and stigma, enhance family relationships and promote family skill building (Asen & Scholz, 2010; Dawson, Baudinet, Tay, & Wallis, 2018; Simic & Eisler, 2015). Some models also specifically aim to intensify treatment, particularly at the early stages (Simic & Eisler, 2015; Wierenga et al., 2018), which has been shown to be a critical time during treatment. Early eating disorder symptom change has been shown to be a robust predictor of end of treatment outcomes across diagnosis, age range, treatment type, and setting (Nazar et al., 2017; Vall & Wade, 2015); hence, the importance of a more intensive intervention, such as MFT, at this stage of treatment.

Given MFT can provide early, intensive support, that focuses on both patient and family factors, it has great potential to improve upon current treatment outcomes either as a stand‐alone or adjunctive intervention. Its use also fits with practice guidelines, which increasingly suggest involving family members in child, adolescent, and adult treatments (Fleming, Le Brocque, & Healy, 2020; Hilbert, Hoek, & Schmidt, 2017; National Institute for Health and Care Excellence (NICE), 2017; Treasure, Parker, Oyeleye, & Harrison, 2021).

Emerging evidence indicates that MFT is associated with improved physical health, a reduction in eating disorder symptoms and improvements in a range of other patient and family factors, such as self‐esteem, quality of life, and caregiver burden (Gelin et al., 2016). Results from the only outpatient randomized controlled trial (RCT) published indicate global outcomes at discharge from family therapy with adjunctive MFT are improved compared to family therapy alone for adolescents with anorexia nervosa (Eisler, Simic, Hodsoll, et al., 2016).

Yet, despite its promise, MFT remains relatively understudied. The heterogeneity of MFT models described and evaluated, as well as the relatively high resource cost and intensity required of some MFT models, makes it difficult to implement and evaluate. Furthermore, MFT is rarely a stand‐alone treatment and large variability exists between studies in the way MFT is delivered, including setting (inpatient, day program, outpatient), treatment duration, and treatment intensity (Gelin et al., 2018), making MFT‐specific findings difficult to generalize.

Given MFT is now widely used in clinical services internationally, a systematic scoping and synthesis of the available data is needed to determine the evidence base and identify gaps for future research. To better understand the impact of MFT on eating disorder treatment outcomes, this study aimed to systematically review and synthesize the available quantitative and qualitative findings. While a review has previously been completed of MFT for a range of psychiatric disorders (Gelin et al., 2018), including eating disorders, this was not exhaustive, missed some important papers (Jewell & Lemmens, 2018), and did not include qualitative data. Specifically, this review has three aims:

1. To review the impact of MFT on the physical and psychological symptoms of eating disorders.
2. To review the impact of MFT on families and caregivers.
3. To review the individual and family experience of receiving MFT.

## 2 METHOD

A systematic scoping review methodology (Peters et al., 2015) was used to explore the existing research into MFT for eating disorders across the age range. This was identified as the most appropriate methodology given the heterogeneity of existing research and the broad aims of this review. This allowed for more descriptive studies that included some outcome data to be included. Current scoping review guidelines (Peters et al., 2020) and Preferred Reporting Items for Systematic Reviews and Meta‐Analyses (PRISMA) extension for scoping reviews guidance (Tricco et al., 2018) were used to conduct this review. The research was reviewed and approved by an institutional review board.

The methodology was initially developed by one author (JB) using the PICOS (population, intervention, comparison, outcome, study design) framework (Methley, Campbell, Chew‐Graham, McNally, & Cheraghi‐Sohi, 2014). Two authors (JB, LD) then independently executed the search strategy, study selection and data extraction. Disagreements were resolved by consensus discussions. Data were reviewed using a parallel‐results convergent synthesis design (Noyes et al., 2019), whereby quantitative and qualitative data were initially analyzed and presented separately; then, synthesized for interpretation of the findings. This was deemed the most appropriate method of initially scoping both the quantitative and qualitative data, as well as synthesizing all available data.

### 2.1 Eligibility criteria

Eligibility criteria for this review are presented in Table 1.

**TABLE 1:** Systematic scoping review eligibility criteria

	Included	Excluded
Publication type	Peer‐reviewed articles Book chapters Dissertations	Conference abstracts Non peer‐reviewed articles
Language	English	Non‐English language
Study objectives	Explicit focus on MFT outcomes Explicit focus on the experience of MFT	Integrated treatment programs where the MFT component is not explicitly reported on or the main focus
Methodology/design	Quantitative Qualitative Mixed methods	Review articles Meta‐analyses Satisfaction, feedback, or acceptability data only (no qualitative data analysis methodology described) Case study design Descriptive quantitative data only (no statistical analyses conducted) Data collection methodology not described
Sample	Any age People with eating disorders Caregivers of people with eating disorders	Clinician only data

### 2.2 Search strategy

Four main databases (PsycInfo, Medline, Embase, CENTRAL) and five grey literature databases (Scopus, Web of Science, OpenGrey, ProQuest Dissertations and Theses Global, EThOS UK Theses) were searched using variations of the terms “eating disorder” and “multi‐family therapy” on 24th June 2021 (see Appendix S1 for exact search terms). References lists of identified articles were then reviewed as a final step for any additional, relevant papers that met the inclusion criteria.

### 2.3 Selection process

**FIGURE 1:**
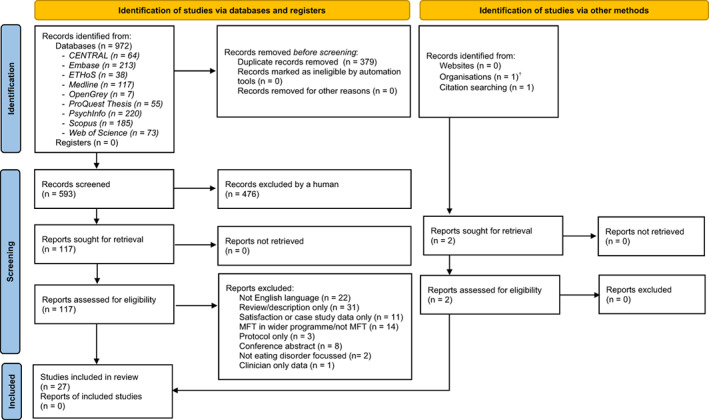
PRISMA flow diagram. ^†^MSc dissertation identified by co‐author (IE) who supervised the work. From: Page MJ, McKenzie JE, Bossuyt PM, Boutron I, Hoffmann TC, Mulrow CD, et al. The PRISMA 2020 statement: an updated guideline for reporting systematic reviews. BMJ 2021;372:n71. doi: 10.1136/bmj.n71. For more information, visit: http://www.prisma‐statement.org/

After completing the initial search, duplicates were deleted, and the remaining titles and abstracts were reviewed. Full‐text citations and reference lists for relevant articles were screened for eligibility before reaching consensus at the included papers in this synthesis (see Figure 1 for PRISMA flowchart). Zotero software was used in this process.

### 2.4 Data extraction, charting, and categorization

All included articles were grouped according to three main categories: type of data generated (quantitative or qualitative), participant age (young person [≤25 years] or adult [≥17 years]), and treatment setting (inpatient/day‐patient/residential or outpatient). Regarding the overlap in how young adults, aged 17–25 years, were categorized: if a program was predominantly child and adolescent focused but included young adults, it was categorized as “young person.” When the participant age range started at 17 years and extended beyond 25 years, this program was categorized as an adult program. No program was exclusively for young adults. Quantitative studies were further charted according to study design (RCT, non‐randomized comparison study, case series).

The data charting forms were developed by JB in consultation with LD to determine which variables to extract. For quantitative studies, data on change in eating disorder symptoms, physical health outcomes, comorbid individual and family factors, and general function data from baseline to discharge (and follow‐up if available) was extracted, as well as effect sizes. MFT program characteristics (intensity and duration) were also extracted. For qualitative data, themes, and sub‐themes were extracted. This was completed by both authors (JB and LD) via an iterative process in repeated consultation. Any missing data were explicitly reported, where applicable. This information was used to inform the narrative synthesis of eligible studies. In line with current scoping review guidance, risk of bias assessment was not completed (Munn et al., 2018; Peters et al., 2020).

## 3 RESULTS

### 3.1 Study selection and characteristics

Nine‐hundred‐and‐seventy‐two papers were initially identified through the systematic literature search. After duplicates were deleted and screening was performed according to the eligibility criteria (Table 1), a total of 27 articles were determined eligible for this review (see Figure 1 for PRISMA flowchart). The total sample reported on who received MFT is 714 (mean age = 18.7 years, range = 11–62, 97% female).

Outcomes from the 27 studies are synthesized below, comprising data generated from one mixed‐method, 17 quantitative, and nine qualitative studies. Three of the quantitative studies also included some qualitative feedback data; however, the data analysis methodology was not adequately reported on to reach the review inclusion criteria. As such, only the quantitative data from these studies are included in this review (Dimitropoulos, Farquhar, Freeman, Colton, & Olmsted, 2015; Mehl, Tomanová, Kuběna, & Papežová, 2013; Wierenga et al., 2018). The only included mixed‐method study was a doctoral dissertation (Salaminiou, 2005), of which most (but not all) of the outcome data were published in a peer‐review journal (Salaminiou, Campbell, Simic, Kuipers, & Eisler, 2017). Both the dissertation and article were identified by the search strategy and included in this review. Data reported in Salaminiou et al. (2017) are reported as such. All remaining quantitative and qualitative data are reported as Salaminiou (2005) henceforth.

Most studies were from Europe (*n* = 18, 67%) and had relatively small sample sizes. Nearly, a quarter of the quantitative studies had 30 participants or less (*n* = 6, 22%) and only two (7%) had a sample size greater than 100. Twenty‐three studies reported on MFT in an outpatient setting (17 young person, six adult) and four on a day‐ or inpatient setting (two young person, two adult). Seven studies compared MFT outcomes to another treatment (five young person, two adult). See Table 2 for a summary of included study characteristics. See Tables 3, 4 and 5 for a summary of the quantitative eating disorder outcomes, quantitative comorbid and family outcomes, and qualitative outcomes, respectively.

**TABLE 2:** Methodologies of included studies

	Young person		Adult		
	OP	I/DP	OP	I/DP	Total
RCT	1	1	0	1	3
Non‐randomized comparison studies	2	1	1	0	4
Case series	7	0	3	0	10
Qualitative	6	0	2	1	9
Mixed‐method	1	0	0	0	1
Total	17	2	6	2	27

Abbreviations: I/DP, inpatient or day‐patient; OP, outpatient; RCT, randomized controlled trial.

**TABLE 3:** Summary of findings for changes in eating disorder symptoms and weight during MFT (*n* = 17)

Author and place	Design	Mean age (*SD*, range)	Sample *N* (% female, race/ethnicity, SES)	Diagnosis	Setting	MFT model	#MFT sessions	Tx length	Baseline data (mean, *SD*)	End of treatment ED data (mean, *SD*)	ES	Dropout (*n*, %) and follow‐up data (time to FU, *n*, % of initial sample^a^)
Young people—outpatient MFT												
RCTs												
Eisler, Simic, Hodsoll, et al. (2016) [UK]	Multi‐Center RCT: FT‐AN (*n* = 82) vs. MFT + FT‐AN (*n* = 85)	15.7 (1.7, 12–20)	167 (F: 91%) (R/E: White 90%, other 4%, missing 6%) (SES: nr)	AN (76%) EDNOS‐R (24%)	Outpatient	Maudsley model (Simic et al., 2021)	10 sessions	12 months	%mBMI: 77.6 (6.3) [MFT] 78.4 (5.8) [FT]	%mBMI: 90.0 (9.1) [MFT] 87.8 (10.1) [FT] Time effect *** Treatment effect *ns*	%mBMI: Time: nr Tx: *z* = .37	Dropout: 18 (11%) 9 (11%) [MFT] 9 (11%) [FT‐AN] FU: 6‐month [18 months post‐randomization] (*n* = 38–60, 22–34%) %mBMI: 90.6 (8.2) [MFT] 85.2 (10.2) [FT] Time effect ***^a^ (ES nr) Treatment effect **^a^ (*z* = .68) EDE‐R: 1.4 (1.7) [MFT] 1.8 (1.4) [FT] Time effect ***^a^ (ES nr) Treatment effect ns^a^ (*z* = .26) Morgan Russel (good and intermediate) outcome: 78% [MFT] 57% [FT] Treatment effect *ns*
									EDE‐R: 2.7 (1.8) [MFT] 2.9 (1.7) [FT]	EDE‐R: 1.8 (1.7) [MFT] 1.4 (1.5) [FT] Time effect *** Treatment effect *ns*	EDE‐R: Time: nr Tx: *z* = .26	
									Morgan Russel outcome (good & intermediate): 8% [MFT] 10% [FT]	Morgan Russel outcome (good and intermediate): 76% [MFT] 58% [FT] Treatment effect*		
Non‐randomized comparison studies												
Gabel et al. (2014) [Canada]	Retrospective chart review comparison: TAU (*n* = 25) vs. TAU + MFT (*n* = 25)	14.1 (2.0, 11–18)	50 (F: 100%) (R/E: nr) (SES: nr)	AN (100%)	Specialist ED service (outpatient, day patient and inpatient)	Maudsley model	nr	12 months	%IBW: 77.7 (nr) [MFT] 79.1 (nr) [TAU]	%IBW: 99.6 (7.3) [MFT] 95.4 (6.8) [TAU] Time effect *sig nr* Treatment effect*	nr	Dropout: nr No FU
									EDE‐Q: 3.1 (1.8) [MFT] nr [TAU]	EDE‐Q: 2.1 (1.4) [MFT]* nr [TAU] Time effect nr Treatment effect nr		
Marzola et al. (2015) [USA]	30‐month follow up: Intensive FT (*n* = 20) vs. MFT (*n* = 54)	14.7 (2.8, nr)	74 (F: 92%) (R/E: Cauc. 92%, other nr) (SES: nr)	AN (60%) EDNOS (40%)	Outpatient	“Intensified FT‐AN”	5 days [40 h]	1 week	%EBW: 86.4 (8.7) [total sample] ED measure: nr	nr [follow‐up data only]	nr	Tx dropout nr [loss to FU: 18 (20%) of eligible participants] FU: 30.9 (20.2, 4–83) month (*n* = 55–74, 60–80%) %EBW: 97.8 (10.1) [MFT] 102.2 (17.5) [I‐FT] Time effect***^a^ (*d* = 1.18) Treatment effect *ns* ED measure: nr [60.8% full remission^b^ 27% partial remission^b^]
Case series												
Dennhag, Henje, & Nilsson, 2019 [Sweden]	Case series	13.9 (1.1, 13–16)	24 (F: 100%) (R/E: Cauc. 100%) (SES: 78.5% parents with uni. Degree/100% fathers and 60% mothers employed full‐time)	AN (37.5%) EDNOS (62.5%)	Outpatient (*n* = 7 commenced MFT while inpatient)	As per Wallin (2007) manual	10.5 days	1 year	BMI: 17.9 (2.0) %EBW: 93.0 (3.7) EDE‐Q (G): 2.9 (1.6)	BMI: 20.1 (2.2)*** %EBW: 101.3 (10.0)** EDE‐Q (G): 2.0 (1.9)* [Full remission: 10 (42%)^c^, No ED dx: 13 (54%)]	*d* = 1.16 *d* = .72 *d* = .56	Dropout: nr No FU
Gelin, Fuso, Hendrick, Cook‐Darzens, and Simon (2015) [Belgium]	Case series	16.0 (1.5, 11–19)	82 (F: 98%) (R/E: nr) (SES: nr)	AN‐R (84%) AN‐BP (11%) BN (5%)	Outpatient	Influenced by Maudsley and Dresden models	21 days	11 months	%EBW: 77.0 (9.8) EDI‐DT: 12.5^d^ (IQR: 5–18)	%EBW: 86.8 (11.2)** EDI‐DT: 4.0^d^ (IQR: 1–11)**	*d* = .93 *r* = .60	Dropout: 7 (9%) FU: 1‐year (*n* = 42, 51%) %EBW: “Continues to increase significantly” EDI: “Stabilize after treatment” [data not reported]
Hollesen, Clausen, and Rokkedal (2013) [Denmark]	Case series	14.9 (1.1, 12–17)	20 (F: 100%) (R/E: nr) (SES: nr)	AN (40%) EDNOS‐R (60%)	Outpatient	Influenced by Maudsley & Dresden models	12 days	1 year (range = 8.4–11.8 months)	BMI: 16.2 (1.4) EDE‐R: 3.2 (1.2) EDI‐DT: 12.5 (5.7)	BMI: 18.4 (1.4)** EDE‐R: 1.8 (1.6)** EDI‐DT: 6.1 (5.7)** [No ED dx: 13 (65%)]	*d* = .63 *d* = 1.0 *d* = 1.13	Dropout: 1 (3%) No FU
Knatz et al. (2015) [USA]	Case series	14.6 (2.9, nr)	40 (F: nr) (R/E: Cauc. 90%, other nr) (SES: nr)	nr	Outpatient	Influenced by Maudsley and Dresden models	5 days [40 h]	1 week	BMI: 17.6 (2.1) ED measure nr	BMI: 20.7 (2.1)** ED measure nr	nr	Dropout: nr No FU
Mehl et al. (2013) [Czech Republic]	Case series	17.7 (2.5, 14–23)	15 (F: nr) (R/E: nr) (SES: nr)	nr	Outpatient	Maudsley model	8	12 months	BMI: 16.9 (1.5) ED measure nr	BMI* (mean and *SD* nr) ED measure nr	nr	Dropout: 2 (13%) No FU
Salaminiou et al. (2017) [UK]	Case series	15.4 (1.8, 11–18)	30 (F: 90%) (R/E: nr) (SES: 50% UK class I/II, 50% class III/IV)^e^	AN (90%) EDNOS‐R (10%)	Outpatient	Maudsley model	9–11 days	9 months	%mBMI: 75.8 (6.5) EDI‐2‐DT: 14.1 (7.1)	%mBMI: 86.1 (8.7)*** EDI‐2‐DT: 9.6 (7.9)**	*d =* 1.4 *d =* .6	Dropout: 2 (7%) No FU
Stewart et al., 2019 [UK] *(study 1 data presented)*	Case series	15.6 (1.4, nr)	50 (F: 98%) (R/E: nr) (SES: nr)	BN (100%)	Outpatient	Maudsley model	14 × 2 h Sessions	4 months	EDE‐Q (SC): 5.3 (1.0) Binge/wk: 7^b^ (range = 0–195) Purge/wk: 10^b^ (range = 0–195) Weight nr	EDE‐Q (SC): 4.81*** Binge/wk: 5^d^ (range = 0–100)** Purge/wk: 3^d^ (range = 0–120)* Weight nr	*r* = −.47 *r* = −.35 *r* = −.29	Dropout study 1: nr Dropout study 2: 2 (4%) [MFT] 18 (21%) [TAU‐no MFT] No FU
Young people—Inpatient MFT												
RCTs												
Geist, Heinmaa, Stephens, Davis, and Katzman (2000) [Canada]	Single‐Center RCT: FT (*n* = 12) vs. Family group psychoed. (MFT, *n* = 13)	14.3 (1.5, 12–17.3)	25 (F: 100%) (R/E: nr) (SES: nr)	AN‐R (76%) EDNOS‐R (24%)	Inpatient [NB: All transitioned to outpatient setting during MFT]	Psychoeducation groups	8 × 90 m fortnightly sessions	4 months	%IBW: 77.2 (11.1) [MFT] 74.9 (9.2) [FT] EDI‐2‐DT: 13.7 (6.2) 11.1 (5.8) [FT]	%IBW: 96.3 (8.2) [MFT] 91.3 (7.3) [FT] Time effect *** Treatment effect *ns* EDI‐2‐DT: 13.3 (7.6) [MFT] 12.3 (7.5) [FT] Time effect *ns* Treatment effect *ns*	*“small to medium”*	Dropout: nr No FU [*59% had concerns about being randomized to new treatment*]
Non‐randomized comparison studies												
Depestele et al. (2017) [Belgium]	Uncontrolled comparison: MFT (*n* = 62) vs. Parent group (*n* = 50)	17.1 (2.2, 14–21)	112 (F: 100%) (R/E: nr) (SES: nr)	AN‐R (41%) AN‐BP (23%) BN (21%) EDNOS (14%) NSSI (62%) BP (53%) Non‐BP (46%)	Inpatient	Maudsley model (Eisler, 2005) *NB: No siblings*	7 (2–3 h sessions)	10 weeks +1 x 6‐month follow‐up session	Weight: nr EDI‐DT: 35.4 (nr) [MFT] 34.0 (nr) [PG]	Weight: nr EDI‐DT: 29.7 (nr) [MFT] 28.1 (nr) [PG] Time effect*** Treatment effect *ns*	nr	Dropout: 13 (12%) 7 (11%) families [MFT] 6 (12%) families [PG] FU: 1 MFT session at 6‐month described (no data reported)
Adult—outpatient MFT												
Case series												
Skarbø and Balmbra (2020) [Norway]	Case series	21.3 (3.5, 17–30)	68 (F: 100%) (R/E: nr) (SES: nr)	AN (76.5%) BN (23.5%)	Outpatient	Adapted from Maudsley and Toronto models	13 days	12 months	BMI: 17.8 (2.1) [total sample] 16.6 (1.5) [underweight group only]	BMI: 18.0 (2.0) [total sample] *ns* 17.4 (1.9)** [underweight group only]	*d* = .1 *d* = .48	Dropout: 5 (7.4%) No FU
Tantillo, McGraw, Lavigne, Brasch, and Le Grange (2019) [USA]	Case series	23 (3.6, 20–31)	10 (F: 100%) (R/E: Cauc. 100%) (SES: nr)	AN (40%) OSFED‐R (60%)	Outpatient	Manualized R4R MFT Group (Tantillo et al., 2020)	16 sessions	26 weeks	BMI: 20.7 (3.3) EDE (global): 2.8 (1.2)	BMI: 21.2 (3.3) *ns* EDE (global): 1.8 (0.7)**	*d* = .16 *d* = .75	Dropout: 0 (0%) FU: 6‐month (*n*, % nr) BMI: 21.5 (3.90) *ns* (*d* = .22)^a^ EDE (global): 1.3 (0.6)** (*d* = 1.41)^a^
Wierenga et al. (2018) [USA]	Case series	24.5 (8.8, nr)	54 (F: 100%) (R/E: Cauc. 85%, other nr) (SES: nr)	AN‐R (52%) AN‐BP (24%) EDNOS‐R (24%)	Outpatient	Neurobiologically informed intensive FT‐AN	5 days [40 h]	5 days	BMI: 18.1 (2.1) EDE‐Q (global): 3.5 (1.3)	BMI: 18.3 (2.0)* EDE‐Q (global): 3.1 (1.5)*	*d* = .10 *d* = .27	Dropout: 1 (2%) FU: >3‐month [EDE data mean = 142.2 (81.5) days / BMI data mean = 228.0 (177.7) days] (n = 28–39, 52–72%) BMI: 19.6 (2.0)*** (*ƞ2* = .41)^a^ EDE‐Q (global): 3.0 (1.5)** (*ƞ2* = .24)^a^
Adult—Inpatient MFT												
RCTs												
Whitney, Murphy, et al. (2012) [UK]	Single‐Center RCT: FT (*n* = 25) vs. 3‐day MFT (*n* = 23)	43.5 [MFT] (14.8, nr) 47.9 [FT] (14.1, nr)	48 (F: 98%) (R/E: nr) (SES: 2% post‐grad degree / 11% employed FT or PT)	AN (100%)	Inpatient	Family day workshops (Treasure, Whitaker, Todd, & Whitney, 2011)	3‐days [FT: 18 h (1–2 h per sessions)]	3 days [FT: Weekly‐fortnightly]	BMI: 13.2 (1.5) [MFT] 13.3 (1.6) [FT] SEED‐AN: 2.6 (0.4) [MFT] 2.5 (0.5) [FT]	BMI: 18.4 (1.8) [MFT] 17.6 (1.9) [FT] Time effect *ns* Treatment effect *ns* Interaction effect* SEED‐AN: 1.8 (0.9) [MFT] 2.0 (1.1) [FT] Time effect *ns* Treatment effect *ns*	BMI: Time: nr Tx: z = .5 SEED‐AN: Time: nr Tx: z = .4	Dropout: 6 (12.5%) 3 (13%) [MFT] 3 (12%) [FT] *NB: One MFT dropout moved to FT group* FU: 3‐year (*n* = 29–44, 60–92%) BMI: 15.8 (2.6) [MFT] 16.8 (2.2) [FT] Time effect *ns* Treatment effect *ns* SEED‐AN: 1.9 (0.9) [MFT] 1.7 (0.7) [FT] Time effect *sig nr* Treatment effect *sig nr*
Non‐randomized comparison studies												
Dimitropoulos et al. (2015) [Canada]	Uncontrolled comparison: FT (*n* = 17) vs. MFT (*n* = 28)	26.2 (7.4, 18–57)	45 (F: 100%) (R/E: nr) (SES: nr)	AN‐R (44%) AN‐BP (56%)	Inpatient (73%) & day program (27%)	Based in Cognitive‐Interpersonal Maintenance Model of AN (Schmidt and Treasure, 2006)^c^	8 × 90 m, sessions	8 weeks	BMI: 15.7 (1.6) [total sample] EDE‐Q (global): 4.1 (1.4) [total sample]	BMI: 20.3 (1.4) [total sample] Time effect** Treatment effect *ns* EDE‐Q (global): 2.5 (1.2) [total sample] Time effect** Treatment effect *ns*	BMI: Time: *ƞ* ^2^ = .88 Tx: nr EDE‐Q: Time: *ƞ* ^2^ = .74 Tx: nr	Dropout: 8 (18%) 5 (17%) [MFT] 3 (18%) [FT] FU: 3‐month (weight and ED data nr, only family FU data)

Abbreviations: ACT, acceptance and commitment therapy; AN, anorexia nervosa; AN‐rd, anorexia nervosa and related disorders; ARFID, avoidant/restrictive food intake disorder; Ax, assessment; BED, binge eating disorder; BMI, body mass index; BN, bulimia nervosa; BN‐rd, bulimia nervosa and related disorders; CBT, cognitive behavioral therapy; CRT, cognitive remediation therapy; DBT, dialectical behavior therapy; DP, day program; ED, eating disorder; ED‐Rs, restrictive eating disorders; EDNOS, eating disorder not otherwise specified; EDNOS‐R, eating disorder not otherwise specified characterised by restriction; EOT, end of treatment; FBT, family‐based treatment; FT‐AN, family therapy for anorexia nervosa; FU, follow up; IBW, ideal body weight; IOP, intensive outpatient program; IP, inpatient; MDT, multi‐disciplinary team; MI, motivational interviewing; OSFED, other specified feeding and eating disorder; OSFED‐R, other specified feeding and eating disorder characterized by restriction; PG, parent group; PHP, partial‐hospitalization program; PMM, predictors, moderators or mediators; RO DBT, radically open dialectical behavior therapy; SES, socioeconomic status; UFED, unspecified feeding and eating disorder.

^a^ Significant testing compares baseline to follow‐up period.

^b^ Median reported instead of mean. Full remission was defined as normal weight (≥95% of expected for sex, age, and height), Eating Disorder Examination Questionnaire (EDE‐Q) global score within 1 *SD* of norms, and absence of binge–purging behaviors. Partial remission was defined as weight ≥85% of expected or ≥95% but with elevated EDE‐Q global score and presence of binge–purging symptoms (<1/week).

^c^ Definition of full remission: at least 95%EBW and EDE global score within 1 *SD* of community norms (Lock, 2018).

^d^ Median reported instead of mean.

^e^ As per Classifications of Occupations 1980, Office of Population Censuses and Surveys, Her Majesty's Stationary Office, London.

*p < .05; **p < .01; ***p < .001.

**TABLE 4:** Summary of the patient, caregiver, and family psycho‐social functioning factors assessed in quantitative studies

	Patient factors			Caregiver factors			Family factors		
	Measure: baseline mean (*SD*)	EOT mean (*SD*)	Effect size	Measure: Baseline mean (*SD*)	EOT mean (*SD*)	Effect size	Measure: Baseline mean (*SD*)	EOT mean (*SD*)	Effect size
Young person—outpatient MFT									
RCT									
Eisler, Simic, Hodsoll, et al. (2016) [UK]	BDI:	BDI:		ECI (negative): 85.6 (nr)	−15.81^a^ [MFT‐AN]**	z = −.52	Nil	Nil	Nil
	23.9 (14.3) [MFT]	−5.9^a^ (nr) [MFT‐AN]***	z = .41		−13.59^a^ [FT‐AN]**	z = −.45			
	25.2 (14.4) [FT‐AN]	−8.8^a^ (nr) [FT‐AN]***	z = −.62	ECI (positive): 28.3 (nr)	1.13^a^ [MFT‐AN] *ns*	z = .13			
	RSES:	RSES:			.42^a^ [FT‐AN] *ns*	z = .05			
	25.1 (6.6) [MFT]	−1.6^a^ (nr) [MFT‐AN] *ns*	z = −.24						
	26.3 (6.9) [FT‐AN]	−.5^a^ (nr) [FT‐AN] *ns*	z = −.08						
Non‐randomized comparison studies									
Gabel et al. (2014) [Canada]	CDI total: 64.8 (15.2) [MFT]	52.9 (18.2) [MFT]*	nr	Nil	Nil	Nil	Nil	Nil	Nil
Marzola et al. (2015) [USA]	Nil	Nil	Nil	Nil	Nil	Nil	Nil	Nil	Nil
**Case series**									
Dennhag et al., 2019 [Sweden]	CGAS: 48.1 (7.3)	61.7 (11.5)***	*d* = .96	EDSIS total (m): 39.9 (12.0) EDSIS total (f): 34.1 (11.9)	26.9 (15.9)*** 23.9 (11.2)***	*d* = .92 *d* = .63	Nil	Nil	Nil
Gelin et al. (2015) [Belgium]	OQ‐45 total: 77.8 (27.3)	46.4 (32.6)***	*r =* .47	Nil	Nil	Nil	Nil	Nil	Nil
Hollesen et al. (2013) [Denmark]	IIP: means, *SD* nr SASB‐Intrex: means, *SD* nr	“Less domineering” *ns* “Less vindictive”* “Less self‐blaming” *ns* “More controlling to mo.” *ns* “More submissive to mo.” *ns* *“*More emancipating to fa.” *ns* “Fa. more ignorant and distant” ns	*d* = .41 *d* = .52 *d* = .52 *d* = .42 *d* = .51 *d* = .35 *d =* .27	Nil	Nil	Nil	Nil	Nil	Nil
Knatz et al. (2015) [USA]	Nil	Nil	Nil	Nil	Nil	Nil	Nil	Nil	Nil
Mehl et al. (2013) [Czech Republic]	RSES^b^: 25.73 (4.20) SOS‐10^b^: 25.73 (9.05)	23.07 (3.75)** 35.13 (9.57)**	nr *η2* = .48	Nil	Nil	Nil	Nil	Nil	Nil
Salaminiou et al. (2017) [UK]	BDI‐II: 27.8 (12.1) RSES: 18.4 (6.9)	17.2 (14.6)** 24.5 (8.3)**	*d* = .8 *d* = .8	BDI‐II (m): 13.9 (6.1) BDI‐II (f): 8.0 (5.6)	9.7 (7.8)* 5.8 (5.6) *ns*	*d* = .7 *d* = .4	Nil	Nil	Nil
Salaminiou (2005) [UK]	As above	As above	As above	SCFI (mother to child)^c^ ‐crit. comms. (m): 1.5 (1.9) ‐pos. rem. (m): .9 (1.1) EOI (m): 1.9 (1.3) ‐warmth (m): 2.0 (1.1) ‐hostility (m): .6 (1.1) SCFI (father to child)^c^ ‐crit. comms. (f): 1.1 (1.5) ‐pos. rem. (f): .64 (.9) ‐EOI (f): 1.2 (1.0) ‐warmth (f): 2.0 (1.1) ‐hostility (f): .4 (.9)	SCFI (mother to child)^c^ ‐crit. comms. (m): .8 (1.4) *ns* ‐pos. rem. (m): 1.4 (1.5) *ns* ‐EOI (m): 1.6 (1.2) *ns* ‐warmth (m): 2.4 (1.2) *ns* ‐hostility (m): .2 (.8) *ns* SCFI (father to child)^c^ ‐crit. Comms. (f): .6 (1.1)* ‐pos. rem. (f): 1.1 (1.1) *ns* ‐EOI (f): 1.1 (1.1) *ns* ‐warmth (f): 2.3 (1.3) *ns* ‐hostility (f): .2 (.7) *ns*	nr	SFI (px): 46.0 (12.9) SFI (m): 41.6 (9.9) SFI (f): 39.8 (10.1)	47.6 (11.1) *ns* 43.6 (9.3) *ns* 40.4 (10.6) *ns*	nr
Stewart et al., 2019 [UK] *(study 1 data presented)*	RCADS (dep.): 73.5 (9.7) RCADS (anx.): 67.3 (11.0) DERS total: 139.5 (13.0)	64.58 (13.23)* 60.26 (13.64)* 110.12 (29.51)**	*d* = −.78 *d* = −.64 *d* = −.64	HADS (dep.): 6.2 (4.0) HADS (anx.): 9.4 (3.5) ECI‐neg.: 84.8 (25.1)	4.68 (3.4)* 7.94 (4.4) *ns* 74.00 (30.9)*	*d* = −.33 *d* = −.31 *d* = −.46	Nil	Nil	Nil
Young person— inpatient MFT									
**RCTs**									
Geist et al. (2000) [Canada]	CDI: 14.0 (4.7) [MFT] 11.8 (6.6) [FT] BSI (px): 1.4 (.9) [MFT] 1.3 (.6) [FT] BSI (m): .6 (.5) [MFT] .7 (.8) [FT] BSI (f): .4 (.3) [MFT] .7 (.7) [FT]	CDI: *ns* 15.4 (4.9) [MFT] *ns* 12.2 (7.4) [FT] BSI (px): *ns* 1.2 (.6) [MFT] 1.2 (.7) [FT] BSI (m): *ns* .6 (.5) [MFT] .6 (.5) [FT] BSI (f): *ns* .3 (.2) [MFT] .4 (.4) [FT]	*“small to medium”*	Nil	Nil	Nil	FAM‐III (px): 50.9 (10.8) [MFT] 48.3 (7.3) [FT]	FAM‐III (px)* 55.8 (7.7) [MFT] 52.2 (8.5) [FT] *(NB: Perceived family functioning worsened)*	*“small to medium”*
Non‐randomized comparison studies									
Depestele et al. (2017) [Belgium]	SIQ‐TR: nr EDES: nr *NSSI: 62% lifetime prevalence*	n/a	n/a	ECI‐neg. (m): 93.9 (3.7) [MFT] 90.6 (4.2) [PG] ECI‐neg. (f): 72.7 (3.7) [MFT] 76.3 (4.6) [PG] ECI‐pos. (m): 29.7 (1.1) [MFT] 29.3 (1.2) [PG] ECI‐neg. (f): 24.2 (1.2) [MFT] 25.4 (1.5) [PG]	ECI‐neg. (m):*** 79.1 (4.0) [MFT] 72.5 (4.7) [PG] ECI‐neg. (f):*** 60.6 (4.5) [MFT] 63.0 (5.3) [PG] ECI‐pos. (m): *ns* 28.7 (1.1) [MFT] 28.4 (1.3) [PG] ECI‐neg. (f): *Ns* 22.0 (1.3) [MFT] 26.9 (1.5) [PG]	nr	FAD‐GF (px): 2.1 (.1) [MFT] 2.1 (.1) [PG] FAD‐GF (m): 2.0 (.1) [MFT] 1.9 (.1) [PG] FAD‐GF (f): 2.0 (.1) [MFT] 2.0 (.1) [PG]	FAD‐GF (px): *ns* 2.1 (.1) [MFT] 2.0 (.1) [PG] FAD‐GF (m): *ns* 1.9 (.1) [MFT] 2.1 (.1)[PG] FAD‐GF (f):* 1.9 (.1) [MFT] 1.8 (.1) [PG]	nr
Adult—Outpatient MFT									
Case series									
Skarbø and Balmbra (2020) [Norway]	Nil	Nil	Nil	Nil	Nil	Nil	Nil	Nil	Nil
Tantillo et al. (2019) [USA]	DERS‐LEA: 15.3 (5.2) DERS‐LAERS: 18.8 (6.9)	12.9 (3.7)* 16.8 (2.3) *ns*	*d* = .49 *d* = .32	Nil	Nil	Nil	Nil	Nil	Nil
Wierenga et al. (2018) [USA]	STAI‐state: 56.2 (12.8) STAI‐trait: 55.3 (10.3)	47.8 (12.3)*** 53.4 (10.0) *ns*	*d* = .68 *d* = .19	Nil	Nil	Nil	FAD‐GF: 2.2 (.59)	2.0 (.53)**	*d* = .36
Adult—Inpatient MFT									
RCTs									
Whitney, Murphy, et al. (2012) [UK]^d^	IIP: 122.5 (39.5) [MFT] 109.8 (30.9) [FT]	IIP: *ns* 117.3 (47.8) [MFT] 110.9 (36.5) [FT]	nr	GHQ: 15.4 (5.9) [MFT] 16.6 (6.1) [FT] ECI‐neg.: 89.6 (30.9) [MFT] 74.7 (32.1) [FT] ECI‐pos.: 29.2 (9.6) [MFT] 25.4 (9.2) [FT] LEE: 72.2 (7.5) [MFT] 75.6 (9.9) [FT]	GHQ:* 14.7 (7.4) [MFT] 15.2 (4.2) [FT] ECI‐neg.: *ns* 79.6 (38.1) [MFT] 65.5 (24.4) [FT] ECI‐pos.: *ns* 30.1 (8.0) [MFT] 25.4 (9.7) [FT] LEE:** 71.8 (8.0) [MFT] 72.9 (8.7) [FT]	nr	Nil	Nil	Nil
Non‐randomized comparison studies									
Dimitropoulos et al. (2015) [Canada]	DCCFS: 15.93 (3.14) [MFT] 14.94 (3.30) [FT]	DCCFS: *Ns* 15.64 (3.02) [MFT] 15.46 (2.89) [FT]	*η* ^2^ = .02	EDSIS total: 35.1 (15.1) [MFT] 35.1 (11.6) [FT] FQ‐crit.: 19.9 (4.7) [MFT] 16.8 (6.2) [FT] FQ‐EOI: 26.2 (4.4) [MFT] 27.1 (4.3) [FT] SPS total: 76.5 (9.4) [MFT] 78.2 (7.1) [FT] DCCFS: 14.3 (3.9) [MFT] 13.4 (2.6) [FT] BDI: 12.6 (9.8) [MFT] 10.5 (7.5) [FT]	EDSIS total:** 22.3 (14.7) [MFT] 18.2 (10.1) [FT] FQ‐crit.:** 16.5 (4.7) [MFT] 15.1 (4.3) [FT] FQ‐EOI:** 24.0 (5.2) [MFT] 24.2 (4.4) [FT] SPS total: *ns* 78.4 (8.8) [MFT] 77.4 (6.3) [FT] DCCFS: *Ns* 14.8 (2.6) [MFT] 13.4 (2.7) [FT] BDI:** 8. (9.9) [MFT] 7.5 (7.5) [FT]	*η2* = .52 *η* ^2^ = .29 *η2* = .45 *η* ^2^ = .02 *η2* = <.01 *η* ^2^ = .25	Nil	Nil	Nil

*Note*: When data for two treatment groups are reported (e.g., MFT and FT) significance values reported are for time effect, not treatment or interaction effect.

List of measures and domains assessed across studies: BDI: Beck Depression Inventory (depression symptoms) (Beck, Steer, & Brown, 1996); BSI: Brief Symptom Inventory (psychological symptoms of psychiatric and medical patients) (Derogatis, 1992); CDI: Children's Depression Inventory (depression symptoms) (Kovacs, 1992); DCCFS: Devaluation of consumers and consumer families scales (perceived discrimination and stigma) (Struening et al., 2001); DERS: Difficulties in Emotion Regulation Scale (emotion regulation) (Gratz & Roemer, 2004); DERS‐LEA: Lack of Emotional Awareness subscale (ability to attend to and acknowledge emotions) (Gratz & Roemer, 2004); DERS‐LAERS: Limited Access to Emotion Regulation Strategies subscale (belief that one can access effective emotion regulation strategies) (Gratz & Roemer, 2004); EDSIS: The Eating Disorders Symptom Impact Scale (impact of caring for person with eating disorder) (Sepulveda, Whitney, Hankins, & Treasure, 2008); ECI: Experience of Caregiving Inventory (negative and positive aspects of caregiving) (Szmukler et al., 1996); FAD: Family Assessment Device‐general family functioning subscale (general family functioning) (Epstein et al., 1983); FQ: Family Questionnaire (expressed emotion‐criticism and emotional overinvolvement) (Wiedemann, Rayki, Feinstein, & Hahlweg, 2002); GHQ: General health Questionnaire (psychological morbidity and distress) (Goldberg & Williams, 1998); IIP: Inventory of Interpersonal Problems (self‐image and perception of interpersonal relations) (Horowitz, Rosenberg, Baer, Ureño, & Villaseñor, 1988); LEE: Level of Expressed Emotion (perceived expressed emotion of caregiver towards person with eating disorder) (Kazarian, Malla, Baker, & Cole, 1990); OQ‐45: Outcome questionnaire (quality of life) (Lambert et al., 1996); RCADS: Revised Child Anxiety and Depression Scale (depression and anxiety symptoms) (Chorpita, Yim, Moffitt, Umemoto, & Francis, 2000); RSES: Rosenberg self‐esteem scale (self‐esteem) (Rosenberg, 1965); SASB‐Intrex: Structural analysis of social behavior‐intrex (perceived interpersonal difficulties, incl. With parents) (Benjamin, 1974:197); SCFI: Standardised Clinical Family Interview (expressed emotion) (Kinston & Loader, 1984, 1986); SFI: self‐report family inventory‐health and competence scale (family functioning) (Beavers, Hampson, & Hulgus, 1985); SIQ‐TR: Self‐Injury Questionnaire‐Treatment Related (self‐harm) (Claes & Vandereycken, 2007); SOS‐10: Schwartz Outcome Scale, Czech version (quality of life) (Dragomirecka, Lenderking, Motlova, Goppoldova, & Šelepova, 2006); SPS: Social Provisions Scale (social support) (Cutrona & Russell, 1987); STAI: Spielberger State–Trait Anxiety Inventory (anxiety symptoms) (Spielberger, Gorsuch, & Lushene, 1970).

Abbreviations: EOT, end of treatment; f, father; FT, family therapy; m, mother; MFT, multi‐family therapy; nr, not reported; NSSI, nonsuicidal self‐injury; PG, parent group; px, patient.

^a^ Mean difference from baseline to EOT (12‐months) presented as means unavailable.

^b^ Measure translated into Czech.

^c^ Expressed emotion scores are from parent towards child.

^d^ Short‐term (3‐month) outcomes.

*p < .05; **p < .01; ***p < .001.

**TABLE 5:** Summary of qualitative findings (*n* = 10)

Author	Design	Mean age	Sample *N* (% female^a^, race/ethnicity^a^, SES)	Diagnosis	Setting	MFT model	#MFT days	Mean length (months)	Analysis methodology/notes	Themes and findings
Young person MFT										
Baumas et al. (2021) [France]	YP and parent focus groups of MFT experience and change mechanisms	Px: 16.3 (2.5, 14–19)	9 [from 4 families] ‐3 Px ‐4 mothers ‐2 fathers (F: 67%) (R/E: nr) (SES: nr)	AN (100%)	Inpatient and Outpatient	Blend of Cook‐Darzens (2007) and “Maudsley model”	10 x 3 h sessions	1 year	Thematic analysis	Main themes (both groups) Difficulty linking patients' evolution and MFT Being in a group has several positive effects MFT improved the family dynamics Criticism of the disparity in the stages of the disease among patients Frustration that family issues could not be discussed in the group Adolescent and parent group specific themes Improvements over the last year Changes in the family emotional tone Parent only group‐specific themes Parents' concerns about the short‐term deleterious effects of MFT on their children Difficulties to cope with the topics discussed in therapy Comparison of multi‐ and single‐family therapy
Duarte (2012) [UK]	Individual interviews and focus groups of YP and parent's experience of MFT for BN	Px: 15.8 (nr, 14–17)	15 [from 9 families] ‐6 Px ‐8 parents ‐1 sibling (F: 100%) (R/E: White brit. 33%, Mixed race 11%, missing 56%) (SES: nr)	BN (% nr) EDNOS‐BP (% nr)	Outpatient	Maudsley model (Stewart et al. 2015)	12	20 weeks	Thematic analysis of groups and individual interviews of YP and parents	Themes Group as a source of support “learning from and with each other” (YP & parent theme) Removed sense of isolation Learning from and with each other Parents/carer's awareness (YP & parent theme) Of own behaviour Of adolescent's experience Family relationships (YP & parent theme) Coping mechanisms/management of BN (YP & parent theme) Improvements in adolescents (parent theme) Moods and feelings BN symptoms A secretive disorder: BN as taboo (parent theme) Communication within the group (YP theme) Limitation in what could be said Space to meet adolescents' needs Challenge within the group
Engman‐Bredvik, Carballeira Suarez, Levi, and Nilsson (2016) [Sweden]	Parent structured interviews on the experiences of MFT	[Px: 14.9 (nr, 12–17)]	12 [from 6 families] ‐0 Px ‐6 mothers ‐6 fathers (F: 100%) (R/E: nr) (SES: nr)	[YP: AN (100%)]	Inpatient and Outpatient	Wallin (2011) model	10 days	1 year	Empirical, psychological, phenomenological method (EPP) (Lundberg, et al., 2007) 1–2 months post‐EOT	Themes: Positive experiences New perspectives Improved family dynamics
Salaminiou (2005) [UK]	YP and parents interviews of the experience of MFT	nr (<18)	34 [from 18 families] ‐16 Px ‐18 mothers ‐10 fathers (F: 94%) (R/E: nr) (SES: 50% UK social class I or II / 50% social class III or IV)^	AN (% nr) EDNOS‐R (% nr)	Outpatient	Maudsley model	9–11 days	9 months	Content analysis of EOT interviews with researcher	Themes Treatment expectations Feelings joining MFT
										2. MFT process A support network for parents A support network for patients Specific interventions MFT environment (practical and relational)
										3. Perceived changed during/due to MFT Perceived changes in the patient Perceived changes in the parent(s)
										4. Future directions Some suggestions
Voriadaki et al. (2015) [UK]	YP and parent experience of MFT process during first 4‐days (focus groups, daily diary writing, rating scales)	nr (nr, 15–16)	15 [from 6 families] ‐5 Px ‐6 mothers ‐4 fathers (F:100%) (R/E: White brit. 80%, Asian brit. 20%) (SES: 100% “social class II or III”)^	AN (100%)	Outpatient	Maudsley model	10 (only experience of first 4 days reflected on)	9 months	Interpretative phenomenological analysis using multiple data sources (focus groups, daily diary writing, rating scales)	Main themes for first 4 days of MFT: Day 1: The similarity in food‐related experiences facilitated awareness of the illness Day 2: Becoming aware of the adolescents' and parents' position and role in relation to illness Day 3: An intense day that revealed the current upsets and future possibilities Day 4: Reflecting on progress achieved and the challenge of recovery
Wiseman, Ensoll, Russouw, and Butler (2019a) [UK]	Focus group and interviews: Caregiver and clinician experience of MFT	[Px: 14.6 (nr, 14–16)]	4 [from 3 families] [family role nr] (F: 100%) (R/E: White brit. 100%) (SES: “Diverse”) [All YP (*n* = 5) declined to participate]	[YP: AN (100%)]	Outpatient	Maudsley model	9 days	7 months	Thematic analysis (Braun & Clarke, 2006)	Main themes identified: The value of offering MFT in a specialist community eating disorder service The set‐up and structure of MFT in a specialist community eating disorder service T he challenges of implementing MFT
Wiseman, Ensoll, Russouw, and Butler (2019b) [UK]	Focus groups and interviews: Caregiver and clinician perspective on how change occurs and how MFT adds to existing treatment pathways	[Px: 14.6 (nr, 14–16)]	4 [from 5 families] ‐0 Px ‐2 fathers ‐1 mother ‐1 g.mother (F: 100%) (R/E: White brit. 100%) (SES: “Diverse”) [All YP (*n* = 5) declined to participate]	[YP: AN (100%]	Outpatient	Maudsley model	10 days	nr	Thematic analysis (Braun & Clarke, 2006)	Sub‐themes of main theme: Mechanisms of MFT for creating recovery‐focused change: The experience of being with other families Family bonding Shifting guilt and shame Intensity of MFT Thinking about AN differently Parental confidence
Adult MFT										
Brinchmann and Krvavac (2021) [Norway]	Patient and families' experience of MFT. Data collected from field observations in 2 MFT groups as well as qualitative group and individual interviews	Px: mean nr (nr, 18–22)	48^b^ [from 12 families] ‐12 Px ‐12 “sets of parents”^c^ ‐9 siblings ‐1 g.mother ‐2 partners] (F:100%) (R/E: nr) (SES: nr)	AN (67%) BN (33%)	Inpatient and (mostly) outpatient	As per Skarbø and Balmbra (2020)	6 x 2–3 day gatherings	1 year	Grounded theory	Main categories: Connectedness and recognition Opening up and sharing
Tantillo, McGraw, Hauenstein, and Groth (2015) [USA]	Focus groups of patient and carer experience of recovery process and emo/beh/ improvement in MFT	Px: 23.4 (6.0, 20–34)	17 [from 10 families] ‐5 Px ‐9 mothers ‐3 fathers (F: 80%) (R/E: Cauc. 82%, Asian Amer. 12%, Latino 6%)^d^ (SES: 80% full time college, 20% working FT)^d^	AN (% nr) EDNOS (% nr)	Outpatient	Relational/motivational MFT group	8 sessions	8 weeks	Content analysis	Themes identified: Recovery is experienced as a long, arduous process marked by many disconnections and intense emotion; MFTG‐RM helped to identify disconnections and renew communication and connections MFTG‐RM helped with identification and expression of emotions MFTG‐RM indirectly helped AN symptom
Whitney, Currin, Murray, and Treasure (2012)) [UK]	60–90 m individual interviews investigating carers experience of FT (*n* = 10) or MFT (*n* = 11)	[Px: 25 (9, 18–53)] Carers: 47 (13, 21–62)	23 [from 15 families] ‐0 Px ‐17 parents ‐4 siblings ‐1 husband ‐1 daughter (F: nr) (R/E: nr) (SES: nr)	[Px: AN (100%)]	Inpatient	Family day workshops (Treasure et al., 2011)	3 days	3 days	Interpretive phenomenological analysis (IPA)	Main themes: Who was involved and what were the experiences of working together? What was involved in the intervention and how was it perceived? When is the intervention presented? Where was the intervention held?
										5. How did the intervention work? Improving communication Making sense of the illness Insight into self, others, and the family Feeling empowered

Abbreviations: AN, anorexia nervosa; BN bulimia nervosa; EDNOS, eating disorder not otherwise specified; EOT, end of treatment; FT, family therapy; MFT, multi‐family therapy; MFTG‐RM, Relational/motivational multi‐family therapy group; nr, not reported; px, patient; SES; socioeconomic status; YP, young person.

^a^ Patient data only.

^b^ Sample size reported is for all participants included in fieldwork and qualitative interviews.

^c^ Counted as *n* = 24 individuals in study total sample above (N = 48).

^d^ Data are for all participants in study (N = 17) as patient‐only data are not reported.

### 3.2 Narrative synthesis

#### 3.2.1 MFT models: Population, setting, intensity, and duration

There was substantial variability in the different types of MFT models described (see Tables 2 and 4). However, when studies were clustered according to age, diagnosis, and setting, more homogeneity emerged. One commonality between most studies was that MFT was an adjunctive treatment. Apart from a stand‐alone 5‐day MFT program described in three studies, all from the same center, MFT was always offered in combination with another form of outpatient treatment (e.g., single‐family therapy) or as part of an inpatient admission.

Outpatient MFT‐AN typically lasted 9–12 months and included between 8 and 21 days of MFT treatment. The only exceptions were the three studies from the same center that offered the stand‐alone 5‐day MFT‐AN model (Knatz et al., 2015; Marzola et al., 2015; Wierenga et al., 2018). Almost all were influenced by the Maudsley Hospital (Simic & Eisler, 2015) and/or Dresden (Scholz & Asen, 2001) models. Outcomes for MFT‐BN were only described in two studies from the same child and adolescent service, which lasted four months (Duarte, 2012; Stewart et al., 2019). Inpatient MFT models for young people were briefer, ranging from 2 (Depestele et al., 2017) to 4 months (Geist et al., 2000). During the latter program, all participants were discharged to outpatient treatment during the course of MFT (Geist et al., 2000).

Adult MFT models were generally much more varied compared to those described for young people. Outpatient adult MFT included a stand‐alone 5‐day model (Wierenga et al., 2018), a 26‐week model (Tantillo et al., 2019), and a 12‐month model (Skarbø & Balmbra, 2020), with the latter specifically targeting a mixed‐diagnostic group of adults with “severe eating disorders.” Two studies described MFT as part of inpatient or day‐patient treatment. One program described was a very brief (3‐day) MFT workshop (Whitney, Currin, et al., 2012; Whitney, Murphy, et al., 2012). The other used an 8‐week program that was offered to those on the inpatient and day‐patient units (Dimitropoulos et al., 2015). Brief MFT was considered more cost effective than family therapy on an adult inpatient unit in one study (Whitney, Murphy, et al., 2012). There were no specific MFT‐BN program for adults identified by the search strategy.

#### 3.2.2 Outcomes: Quantitative results

##### Randomized controlled trials

Three studies used an RCT design, all of which compared MFT to a version of single‐family therapy (see Table 2). One investigated MFT‐AN for young people in an outpatient setting (Eisler, Simic, Hodsoll, et al., 2016), one for MFT for young people on an inpatient unit (Geist et al., 2000), and one for adult inpatient MFT (Whitney, Murphy, et al., 2012). See Table 2 for details.

The largest study (*N* = 167), and only multi‐center trial identified by the search strategy, randomized young people (age range = 12–20) to 12 months of outpatient FT‐AN alone or FT‐AN plus 10 days of MFT‐AN (Eisler, Simic, Hodsoll, et al., 2016). No significant differences between groups at baseline were observed. Regardless of the treatment arm, significant improvements in global outcomes, weight, eating disorder psychopathology, and mood, as well as the negative aspects of caregiving were reported. Participants randomized to receive MFT‐AN also had better global outcomes at end of treatment, using the Morgan Russel outcome criteria (Russell, Szmukler, Dare, & Eisler, 1987), compared to those who received FT‐AN alone. Seventy‐six percent had a Good or Intermediate outcome in the MFT group compared to 58% in the FT‐AN group (Eisler, Simic, Hodsoll, et al., 2016). This difference was no longer statistically significant at 6‐month follow‐up (18‐months post randomization); however, the MFT group continued to have significantly higher %mBMI (MFT‐AN group = 91% vs. FT‐AN group = 85%, respectively). Self‐report self‐esteem did not change between baseline and end of treatment in either study arm, although the authors note that baseline scores were within the normal range, suggesting a ceiling effect (Eisler, Simic, Hodsoll, et al., 2016).

The remaining two RCTs identified were both much smaller and conducted on inpatient units. Geist et al. (2000) randomized adolescents (*N* = 25) to receive either single‐family therapy or MFT as part of their inpatient treatment package. No baseline differences between the groups were reported. Treatment in both arms was associated with physical health and eating disorder symptom improvement; however, no differences were reported in weight, eating disorder symptoms, or family functioning outcomes between the two treatments (Geist et al., 2000). Contrary to findings from the Eisler, Simic, Hodsoll, et al. (2016) RCT, no changes in symptoms of depression or severity of general psychopathology were reported (Geist et al., 2000). Notably, self‐report family functioning significantly deteriorated in both treatments, indicating an acknowledgement of more family psychopathology at the end of treatment.

On an adult inpatient unit, Whitney, Murphy, et al. (2012) randomized participants (*N* = 48) to either 18 hours of weekly/fortnightly single‐family therapy or a 3‐day MFT intervention during their admission. They reported a significant treatment by time interaction effect. Post hoc comparisons showed that participants who received MFT had higher BMI at 6‐month follow‐up, but lower BMI at 36‐month follow‐up. However, these did not reach statistical significance. Across both treatments, a significant reduction in expressed emotion and improvement in caregiver general wellbeing was also observed; however, neither the negative nor positive aspects of caregiving significantly changed. Furthermore, no differences between the treatments were reported on any other individual or family outcome measure at short (3‐month) and long (3‐year) term follow‐up, potentially emphasizing the general benefits of family involvement, rather than any MFT‐specific benefits.

##### Non‐randomized comparison studies

Two outpatient (both young person) and two inpatient studies (one young person, one adult) compared outcomes following MFT to another type of treatment using a non‐randomized design (see Table 2 for details).

In a retrospective chart review of treatment response in a specialist child and adolescent service (*N* = 50), Gabel et al. (2014) compared those who received MFT as part of their treatment package with age‐matched controls who received treatment as usual, defined as medical monitoring, nutrition therapy including meal plans, pharmacological treatment as required, psychoeducation, individual, and/or mental health therapy. Those who received MFT in addition to treatment as usual had significantly higher mean weight after 12 months than those who only had treatment as usual (99.6%IBW vs. 95.4%IBW, respectively). Outcomes for the sub‐group who received MFT showed significant improvements in eating disorder and depression symptoms, although these changes were not compared to the control group outcomes. There were no differences between the two groups at baseline.

Marzola et al. (2015) also conducted a retrospective chart review. They compared outcomes at follow‐up for two different 5‐day versions of intensive outpatient family therapy for young people; intensive single‐family, and MFT. End‐of‐treatment (5 days) outcomes were not compared; however, each is associated with eating disorder symptom improvements and reported separately elsewhere (Knatz et al., 2015; Rockwell, Boutelle, Trunko, Jacobs, & Kaye, 2011). At follow‐up (mean = 30.9 months, *SD* = 20.2) both treatments continue to be associated with improvements, although no differences between groups with respect to %mBMI, global outcomes and need for higher levels of care (inpatient or residential) are reported (Marzola et al., 2015). Of note, the MFT group were significantly younger (16.4 vs 19.2 years), had a shorter time to follow‐up (22.5 vs. 53.4 months), and more were undergoing treatment at follow‐up (58% vs. 32%), making comparisons tentative.

In the inpatient context, the benefits of MFT over other types of intervention are less clear. Depestele et al. (2017) compared adjunctive MFT with or without young person involvement (i.e., MFT vs. parent groups). Treatment allocation was not randomized and was dependent on the time of admission to the unit with the type of intervention offered switching every 6 months. At end of treatment, both groups reported a significant improvement in eating disorder symptoms and some aspects of family functioning, although no differences were reported according to treatment type. Specifically, fathers reported improved general functioning and young people reported improved problem solving, whereas mothers reported decreased problem solving on the Dutch version of the Family Assessment Device (FAD) (Epstein, Baldwin, & Bishop, 1983; Wenniger, Hageman, & Arrindell, 1993). No differences in behavioral control, affective involvement, or affective responsiveness were reported after MFT (Depestele et al., 2017). At baseline, the groups did not differ with regard to eating disorder symptoms or other family factors, although the MFT group was significantly older (17.4 vs. 16.6 year) and fewer reported engaging in non‐suicidal self‐injury (51.6 vs 74.0%). These differences were controlled for in analyses.

Using an uncontrolled pilot study design (*N* = 45), Dimitropoulos et al. (2015) compared 8 weeks of either single‐family therapy or MFT for adults receiving day‐ or inpatient treatment. Treatment was assigned non‐randomly based on MFT availability, which was offered four times per year, and no baseline differences in patient or family characteristics were observed between the two treatment groups. At the end of treatment, Dimitropoulos et al. (2015) reported that a range of individual (BMI, eating disorder symptoms, perceived stigma) and caregiver factors (perceived burden, expressed emotion, and caregiver symptoms of depression) all significantly improved. These changes were either maintained (caregiver burden) or continued to improve (expressed emotion and caregiver depressive symptoms) at a 3‐month follow‐up. Nevertheless, the level of perceived social support and impact of stigma for caregivers did not change across treatment or follow‐up period (Dimitropoulos et al., 2015). Furthermore, no differences between interventions were reported on any individual (BMI and eating disorder psychopathology) or caregiver factors (perceived burden, expressed emotion, perceived social supports, and stigma) (Dimitropoulos et al., 2015).

##### Case series

###### Physical health and eating disorder symptomatology

MFT‐AN was associated with significant improvements in weight, regardless of age or treatment setting (see Table 2). Only one study did not report a significant improvement in weight during MFT; however, participants in this study started treatment within the healthy range (mean BMI = 20.7, *SD* = 3.3), which was maintained during treatment (Tantillo et al., 2019). Significant improvements were also reported in eating disorder psychopathology, including binge‐purge symptoms, by every study that measured it, irrespective of age, setting or instrument used (see Table 2).

###### Comorbid symptomatology

Outpatient MFT‐AN for young people was associated with a significant reduction in symptoms of depression from baseline to discharge (Salaminiou et al., 2017). Salaminiou et al. (2017) reported that symptoms of depression reduced from just below the “severely depressed” range to within the “mild” range after 6 months of MFT‐AN. Similarly, self‐report symptoms of both depression and anxiety significantly reduced during outpatient MFT‐BN, although, parent reports of their child's symptoms did not reveal significant changes (Stewart et al., 2019).

The only adult MFT case series to investigate comorbid symptoms found that state, but not trait, anxiety reduced during a 5‐day MFT week (Wierenga et al., 2018). Change in symptoms of depression was not investigated in any adult MFT study in this review.

###### Broader individual functioning and well‐being

Several studies assessed broader symptoms of general well‐being in addition to eating disorder symptom change (see Table 3). Outpatient MFT‐AN for young people was associated with significant improvements in quality of life (Gelin et al., 2015; Mehl et al., 2013), self‐perception and self‐image (Hollesen et al., 2013), and self‐esteem (Mehl et al., 2013; Salaminiou et al., 2017). MFT‐BN for adolescents was associated with significant improvements in emotion regulation capacity (Stewart et al., 2019).

Regarding MFT for adults, at the end of treatment, patients reported significant improvements in emotional awareness but no change in emotion regulation strategies (Tantillo et al., 2019). In the inpatient context, difficulties with interpersonal functioning did not change from baseline to short‐ and long‐term follow‐up (Whitney, Murphy, et al., 2012).

###### Family functioning

In outpatient MFT‐AN for young people, Salaminiou (2005) found that family functioning did not change during 6 months of MFT, although the author noted that mean scores at baseline were mid‐ranged, indicating adequate family functioning. For adults who attend a 5‐day MFT, a significant improvement in general family functioning was reported (Wierenga et al., 2018). See Table 3 for further details.

###### Parent and caregiver factors

Outpatient MFT‐AN for young people was associated with a range of caregiver/parent improvements. By the end of treatment, caregiver burden and most negative impacts of the illness significantly reduced in one study (Dennhag et al., 2019). Perceived caregiver isolation was the only aspect that did not change during MFT (Dennhag et al., 2019). In another study, parental mood improved (Salaminiou et al., 2017). This improvement was significant for mothers, but not fathers, however, baseline maternal and paternal scores were within the normal range, suggesting a floor effect (Salaminiou et al., 2017). In the same study, adjusted regression analysis revealed change in parental depressive symptoms across treatment was not associated with young person percentage median Body Mass Index (%mBMI) outcome at end of treatment (Salaminiou, 2005). Following outpatient MFT‐BN, caregiver burden and parental mood also significantly improved in one study, although level of anxiety did not (Stewart et al., 2019).

The impact of MFT on expressed emotion (critical comments, positive remarks emotional overinvolvement, warmth, and hostility) is mixed. Paternal critical comments significantly reduced from baseline to 6 months in one study (Salaminiou, 2005); however, all other aspects of maternal and paternal expressed emotion towards the child did not change during 6 months of treatment. Salaminiou (2005) also measured level of expressed emotion between parents, as one marker of how well the parental dyad was functioning. Again, no change was observed, except for warmth from mothers towards fathers, which significantly increased (Salaminiou, 2005). Furthermore, adjusted regression analysis revealed that a reduction in paternal criticism and an increase in emotional overinvolvement during MFT‐AN was associated with improved young person %mBMI at end of treatment in the same study (Salaminiou, 2005).

Dennhag et al. (2019) also explored whether baseline and change in caregiver factors during MFT were associated with young person outcomes. At baseline, maternal level of guilt was associated with poorer end‐of‐treatment eating disorder symptom outcomes. Furthermore, increased paternal social isolation and perceived burden of dysregulated behaviors was also associated with poorer physical health outcomes for the young person at end of treatment (Dennhag et al., 2019). Regarding parental change over treatment and its association with outcome, regardless of role in the family, decreases in parental perceived isolation was associated with improved young person physical health and general functioning at end of treatment (Dennhag et al., 2019).

###### Outcomes at follow‐up

Follow‐up data are reported in three adult MFT case series. They indicate that end‐of‐treatment improvements are generally maintained or improved upon at follow‐up. Wierenga et al. (2018) reported that weight continued to significantly increase, with mean BMI within the healthy range (mean = 19.6, *SD* = 2.0), and eating disorder symptom improvement was maintained at follow‐up from their stand‐alone 5‐day MFT program (mean duration to follow‐up = 4.7–7.6 months [varies depending on measure]). Similarly, Tantillo et al. (2019) found that participants maintained their weight and eating disorder symptoms continued to improve at 6‐month follow‐up from their 26‐week program (see Table 2 for further details). In addition, emotion regulation capacity was either maintained or continued to improve during the follow‐up period (Tantillo et al., 2019). One case series with young people reported that weight continued to significantly improve, while the quality of life and eating disorder symptoms stabilized at 1‐year follow up; however, no data were reported (Gelin et al., 2015).

###### Non‐completion rates (all study designs)

MFT non‐completion, also referred to as dropout, is typically reported to be low and is an often‐stated benefit of the treatment (Gelin et al., 2018). Twelve of the 17 quantitative studies reported dropout rates, which ranged from 0% to 17%. Six of these studies reported dropout rates below 10% (see Table 3 for details). Data are presented here synthesized, rather than by study design, to provide a better overview of all available data.

#### 3.2.3 Experience of MFT: Qualitative data

Qualitative data are reported in 10 studies: seven reporting on the experience of MFT for young people and their family members, and three on MFT for adults and their family members. Most commonly, data were generated from individual or focus group interviews and responses analyzed using thematic or content analysis. See Table 3 for further details. The total sample reported on consisted of 47 people with eating disorders (30 young people and 17 adults) and 140 caregivers (120 parents, 14 siblings, three partners, two grandparents, and one adult patient's child). Several papers also reported on clinician experience of MFT (Brinchmann et al., 2019; Wierenga et al., 2018; Wiseman et al., 2019a, 2019b), which is not reported here as it is beyond the scope of this review. Of note, the majority of qualitative data are generated from the family and caregiver perspective. Only six studies (four young persons, two adults) included patients in their sample, and two studies, which appear to use the same sample, noted that they attempted to recruit young people but all declined (Wiseman et al., 2019a, 2019b).

Qualitative studies were initially reviewed separately according to MFT target population (young person or adult) and setting (outpatient or day/inpatient) and were intended to be presented separately. However, due to large overlap in participants' experiences, the data are synthesized and presented together.

Across all studies, there was a common finding that MFT is experienced as both helpful and challenging with similar experiences described for adults and young people for both MFT‐AN and MFT‐BN. From data generated through observation, interviews and focus groups collected during and after treatment, there was a sense by most participants that MFT helped the family to view the eating disorder symptoms in new ways (Baumas et al., 2021; Duarte, 2012; Salaminiou, 2005; Voriadaki, Simic, Espie, & Eisler, 2015), take on new perspectives (Duarte, 2012; Engman‐Bredvik et al., 2016; Tantillo et al., 2015;Whitney, Currin, et al., 2012; Wiseman et al., 2019b), gain new skills (Duarte, 2012; Tantillo et al., 2015) and feel more empowerment (Engman‐Bredvik et al., 2016; Salaminiou, 2005). Together, this helped people, particularly parents/caregivers, feel less guilty, scared, and anxious (Whitney, Currin, et al., 2012) and feel more confident (Whitney, Currin, et al., 2012; Wiseman et al., 2019b). In two studies, adult patients and carers noted that MFT helped them to open up and share their experiences (Berit Støre Brinchmann & Krvavac, 2021; Tantillo et al., 2019).

A common theme across several studies was that MFT led to a shift in the quality of family connection and dynamics (Baumas et al., 2021; Berit Støre Brinchmann & Krvavac, 2021; Duarte, 2012; Tantillo et al., 2015; Wiseman et al., 2019b). Commonly, parents/caregivers felt MFT provided a new support network that helped people in all family roles feel less alone and isolated (Duarte, 2012; Engman‐Bredvik et al., 2016; Salaminiou, 2005; Tantillo et al., 2015; Wiseman et al., 2019b), which was also echoed by some patients, albeit fewer (Duarte, 2012; Salaminiou, 2005). There was value placed on being able to observe and learn from other families who had similar experiences (Duarte, 2012; Whitney, Currin, et al., 2012; Wiseman et al., 2019b). In one study, participants struggled to differentiate the contribution that MFT made to their overall treatment compared to other elements of their treatment program (Baumas et al., 2021).

The challenging aspects of MFT were multifaceted. There were concerns by some about the potential for unhelpful comparisons to be made, the fact that individual family needs could not always be addressed, and that it was difficult at times to manage disparities regarding the different rates of recovery for each person in the group (Baumas et al., 2021). Of note, comparisons were also sometimes seen as helpful as they helped people feel validated and less isolated (Voriadaki et al., 2015).

A minority of parents/caregivers mentioned concerns that the group may set recovery backwards or that the patients may share unhelpful eating disorder “tricks” (Baumas et al., 2021; Salaminiou, 2005). The intensity of the group was also mentioned by some participants as both helpful and exhausting (Voriadaki et al., 2015; Whitney, Currin, et al., 2012; Wiseman et al., 2019b).

To understand changes in the patient experience during MFT, Voriadaki et al. (2015) collected data at different time points over four consecutive MFT days. They found that participants tended to move from anxiety and apprehension about attending, to noticing similarities and then feeling more settled. This helped people to become more aware of the illness and the role in it played in their relationships. By the end of the 4 days, the focus was shifted towards future coping and reflecting on progress. This matches data reported by Wiseman et al. (2019a) who specifically investigated the way family members perceived change to occur in MFT. No young people consented to participate in this study; however, parents perceived the treatment mechanisms to include the increased intensity, the experience of being with other families, family bonding, shifting of guilt and blame, improved parental confidence, and understanding the illness differently (Wiseman et al., 2019a).

#### 3.2.4 Meta‐synthesis

Taken together, MFT is both perceived as helpful and leads to a wide range of improvements. It is also associated with several challenges for different participants at different time points. The benefits regarding eating disorder symptoms and other individual and family factors are reported by most, more so by family members than patients, and are reflected in robust quantitative and qualitative findings of eating disorder symptoms and physical health improvements by the end of treatment and often at follow‐up.

As might be expected of any eating disorder treatment, MFT is also challenging, which is reflected in the more mixed family and caregiver quantitative and qualitative data. There are difficulties associated with the group process, such as concerns about the comparison that comes from being in a group, the intensity of MFT, and the realization of needing to try new things. However, these were balanced by a reduction in perceived isolation and support from the group. The low dropout rate further supports that the group process is engaging and acceptable.

The work required of participants during MFT coupled with the increased support afforded by coming together does yield rewards. Eating disorder symptoms and physical health do consistently improve, but the difficulty and anxiety associated with reaching these changes are clearly reflected in the experiences, and more mixed quantitative findings, particularly regarding family factors. Nevertheless, while qualitative data suggest a key benefit of MFT is a reduction in isolation and an increase in solidarity, this may not generalize beyond the MFT group itself. Quantitative findings from one study indicate that self‐report perceived social supports, isolation, and stigma more broadly do not significantly change during MFT.

One notable finding is that MFT can be described by the patient as more helpful for other family members, yet improvements are more consistently observed for the patient themselves. This fits with findings from one study, in which the majority of parents reported that the benefits of MFT for the patient came indirectly via themselves as parents (Salaminiou, 2005). This perceived disparity in helpfulness may also reflect the stage of recovery of participants at the time of data collection (typically during or at the end of treatment). What is perceived as unhelpful or challenging in the moment may be perceived as helpful and needed with hindsight. Salaminiou (2005) found that participants experienced some exercises to be both difficult and helpful, and vice versa, bringing into question the usefulness of the dichotomous helpful/unhelpful divide when discussing MFT (and potentially other psychological treatments). Furthermore, there was relatively good agreement between participants regarding which MFT activities were perceived as helpful, yet little agreement on what was perceived as unhelpful. This further highlights how specific and idiosyncratic unhelpful aspects may be to each individual.

This disparity also underscores the important role that family members have in the recovery process and MFT's capacity to support the entire system, not just the patient. It emphasizes the need for MFT (and potentially other interventions) to continue to include and support family members, given their own levels of need and the mixed outcomes for parents/caregivers specifically. Of note, there are no data comparing the experience of MFT to the experience of other treatments, making it difficult to determine whether these processes are unique to MFT or common across all eating disorder interventions that involve family members. Qualitative data collected at different time points are also needed to understand whether the patient perspective shifts at longer follow‐up.

## 4 DISCUSSION

MFT has been used in clinical practice for decades (Gelin et al., 2018; Gelin et al., 2016) and practice guidelines often recommend including and supporting family members in eating disorder treatments (Hay et al., 2014; Hilbert et al., 2017; NICE, 2017). The current review highlights that MFT is associated with improvements in a range of individual and family factors. However, the evidence base is relatively small, with most studies underpowered and large heterogeneity in the MFT models tested.

Regarding the first aim of this study, it can be concluded that MFT is associated with physical and psychological improvement for people with eating disorders, across the age range, treatment setting, and diagnoses. This was almost unanimously reported across all studies, often with medium and large effect sizes. However, with the exception of a stand‐alone 5‐day model, all other MFT models were adjunctive treatments. As such, the unique benefits of MFT cannot be ascertained from the available data.

When MFT outcomes are compared to other types of treatment, the findings are mixed. There is evidence from two studies that 12 months of MFT alongside single‐family therapy or treatment as usual may lead to better global outcomes and higher weight at follow‐up for young people in an outpatient setting. This finding was reported in the largest study and only outpatient RCT that was identified in this review, meaning more weight could arguably be given to this finding. However, most other studies that included a comparison group found outcomes following MFT were equivalent to other treatments, both in outpatient and inpatient settings and across the age range. Furthermore, outcomes following intensive versions of family therapy have similar outcomes, regardless of whether it is provided in the single‐ or multi‐family format. One potential benefit of MFT was its cost‐effectiveness as an adjunctive adult inpatient treatment, although more data is needed here to form any firm conclusions.

Regarding the impact of MFT on individual and family/caregiver factors, the data highlights a range of benefits for all involved. For the patient, it is associated with improvements in symptoms of depression and anxiety, self‐esteem, quality of life, and facets of emotion regulation capacity. However, MFT does not seem to be associated with improvements in perceived interpersonal problems or stigma.

Family members also report improvements in their own symptoms of depression, improvements in the negative aspects of caregiving, general well‐being, and some changes in level of expressed emotion, although these data are mixed. This fits with evidence that not all aspects of expressed emotion may be as relevant for adolescents as they are with adults. A recent review found that parental emotional overinvolvement with adolescents was not associated with problematic symptoms or behaviors across a range of mental health diagnoses, and may even have some benefits (Rienecke, 2020). No changes were reported in the positive experiences of caregiving, level of caregiver anxiety, or perceived stigma and social supports. Data regarding changes in general family functioning are more mixed with some studies reporting improvements, others no change, and one a deterioration in functioning.

These findings match the data on the experience of MFT well. These data show that MFT is generally valued, albeit with a fair degree of variability. Benefits are reported by most, especially the increase in perceived support by being in a group with people who have had similar experiences. The process can be exhausting and anxiety provoking, as might be expected of any psychological treatment, and may be part of what makes the treatment effective. Some participants raised concerns about the inevitable comparison that comes from being in a group and the realization of needing to try new things. However, available data on the process of change during MFT suggests these anxieties are alleviated quickly and by the third MFT day participants are more settled and perceived support from the group has increased. Given the experience of MFT can be intense and anxiety provoking, it is unsurprising that some aspects of caregiving and family functioning remain unchanged. Whether MFT experiences and learning lay a foundation for future benefits is yet to be determined and no data are available to report on this.

Despite the many benefits of MFT, the current review highlights some key areas for future research. Generally, the included studies were uncontrolled with small sample sizes, meaning most are likely underpowered. Even the RCTs tended to be small, with two of the three identified in this review having sample sizes of less than 50 participants. Furthermore, given most MFT models were adjunctive, the specific contribution and cost‐effectiveness of adding MFT to other treatments remain unclear. Future studies are needed that examine the unique contribution made by MFT, both regarding outcomes and the experience of treatment, and whether the additional resources required of families and services are worth the benefit.

### 4.1 Limitations

There are several important limitations to this review. First, only English language studies were reviewed, and the publication type was limited to peer‐reviewed journal articles, book chapters, and dissertations (not conference abstracts). Furthermore, this is not an exhaustive review of MFT treatment models. Many theoretical and descriptive papers exist outlining MFT models that vary from those described here, without reporting any data or methodology for data collection.

Regarding the papers reviewed, the most notable are the small sample sizes and uncontrolled nature of study methodologies. Even though three RCTs were identified, two had sample sizes below 50. Similarly, sample sizes for qualitative studies were also often small, with the voice of the person with the eating disorder limited or missing. Furthermore, there was a lack of diversity across the studies. The sample reported on was predominantly white and female with very little socioeconomic data reported. This makes interpretation of the data and conclusions from this review very tentative.

Finally, it is very hard to determine treatment response for people with bulimia nervosa and other eating disorder presentations. There was no data for adult MFT‐BN and very limited data for young people. MFT‐BN findings are very preliminary.

## 5 CONCLUSIONS

The current review suggests MFT is an effective treatment for anorexia nervosa and leads to improvements in individual, as well as some caregiver and family factors. The most robust finding is for young people seen in an outpatient setting. When added to single‐family therapy, MFT may enhance outcomes compared to single‐family therapy alone, although replication studies are needed. Several benefits afforded by MFT appear unique to the multi‐family context; however, the impact of these benefits (e.g., increased support) cannot be determined from the current review. When compared to other types of treatment, MFT is generally non‐inferior, although it is typically an adjunctive treatment, making it difficult to determine its value alone. Future studies are needed that specifically investigate the unique contribution and cost‐effectiveness of MFT compared to other treatments. The evidence base also needs strengthening with higher quality studies with larger sample sizes and more diverse eating disorder presentations. Study designs that consider patient and family preferences, and previous treatment history are also needed, as well as clearer indication criteria for MFT and extended guidelines for the use of MFT in stepped care approaches.

## CONFLICT OF INTEREST

Ulrike Schmidt is supported by a National Institute of Health Research (NIHR) Senior Investigator Award. She receives salary support from the NIHR Mental Health Biomedical Research Center (BRC) at the South London and Maudsley NHS Foundation Trust and King's College London (KCL).

## Supporting information


**Appendix** S1: Supporting Information

## Data Availability

Data sharing is not applicable to this article as no new data were created or analyzed in this study
