# Supplementary material for: Multi‐family therapy for eating disorders: A systematic scoping review of the quantitative and qualitative findings
Source: Int J Eat Disord. 2021 Oct 20;54(12):2095–120. doi: 10.1002/eat.23616 (PMC9298280; doi:10.1002/eat.23616)
Supplement: Supplementary file 1 — Appendix S1: Supporting Information [file EAT-54-2095-s001.pdf]

Supplementary Material 1 | Search Terms and Strategy

Main Databases: Embase, Medline, PsycInfo, Central
Search Date: 24th June, 2021

Concept	Databases: PsycInfo, Embase, Medline	Database: CENTRAL (Cochrane)
Eating Disorders	1. Eating Disorders/ (explode)	1. Eating Disorders/ (explode)
	2. Eating disorder*.mp	2. Eating disorder*
	3. (feeding and eating disorder*).mp	3. feeding and eating disorder*
	4. (feeding adj3 eating disorder*).mp	4. feeding NEAR/3 eating disorder*
	5. Appetite disorder*.mp	5. Appetite disorder*
	6. feeding disorder*.mp	6. feeding disorder*
	7. (eating and feeding disorder*).mp	7. eating and feeding disorder*
	8. (eating adj3 feeding disorder*).mp	8. eating NEAR/3 feeding disorder*
	9. (restrict* adj3 eat*).mp	9. restrict* NEAR/3 eat*
	10. Anorexi*.mp	10. Anorexi*
	11. Bulimi*.mp	11. Bulimi*
	12. ARFID.mp	12. ARFID
	13. Avoidant restrictive food intake disorder.mp	13. Avoidant restrictive food intake disorder
	14. Bing*.mp	14. Bing*
	15. Purg*.mp	15. Purg*
	16. Night eating syndrome.mp	16. Night eating syndrome
	17. (Rumination adj3 disorder*).mp	17. Rumination NEAR/3 disorder*
	18. (Rumination adj3 syndrome).mp	18. Rumination NER/3 syndrome
	19. Pica.mp	19. Pica
	20. allotriophagy.mp.	20. allotriophagy
	21. geophag*.mp	21. geophag*
	22. (Other specified feeding and eating disorder).mp	22. Other specified feeding and eating disorder
	23. OSFED.mp	23. OSFED
	24. (Unspecified feeding and eating disorder).mp	24. Unspecified feeding and eating disorder
	25. UFED.mp	25. UFED
	26. (Eating disorder not otherwise specified).mp	26. Eating disorder not otherwise specified
	27. EDNOS.mp	27. EDNOS
Multi-Family Therapy	28. Multifamil*	28. Multifamil*
	29. Mult*?famil*	29. Mult*famil*
	30. Mult* adj2 famil*	30. Mult* NEAR/2 famil*
	31. Famil* adj1 group*	31. Famil* NEAR/1 group*
	32. MFT	32. MFT
	33. MFTG	33. MFTG
	34. MFG	34. MFG
	35. MFGT	35. MFGT

Search	(#1 OR #2 OR #3 OR #4 OR #5 OR #6 OR #7 OR #8 OR #9 OR #10 OR #11 #12 OR #13 OR #14 OR #15 OR #16 OR #17 OR #18 #19 OR #20 OR #21 OR #22 OR #23 OR #24 OR #25 OR #26 OR #27) ***AND*** (#28 OR #29 OR #30 OR #31 OR #32 OR #33 OR #34 OR #35)

Abbreviations: mp=title, abstract, heading word, table of contents, key concepts, original title, tests & measures, mesh

Grey Literature Databases: ETHos UK Theses Online, OpenGrey.eu, ProQuest Dissertations and Theses Global, SCOPUS, Web of Science
Search Date: 24th June, 2021

Database	Search Terms
ETHoS UK Theses online	Multifamily OR Multi-family OR Multi family OR Multiple-family OR Multiple family
OpenGrey.eu	(Eating disorder* OR Feeding and eating disorder* OR feeding NEAR/3 eating disorder* OR Appetite disorder* OR feeding disorder* OR eating and feeding disorder* OR eating NEAR/3 feeding disorder* OR restrict* NEAR/3 eat* OR anorexi* OR bulimi* OR arfid OR "Avoidant restrictive food intake disorder" OR bing* OR purg* OR "Night eating syndrome" OR Rumination NEAR/3 disorder* OR Rumination NEAR/3 syndrome OR pica OR allotriophagy OR geophag* OR "Other specified feeding and eating disorder" OR osfed OR "Unspecified feeding and eating disorder" OR ufed OR "Eating disorder not otherwise specified" OR ednos) ***AND*** (multifamil* OR mult*famil* OR mult* NEAR/2 famil* OR famil* NEAR/1 group* OR mft OR mftg OR mfg OR mfgt)
ProQuest Dissertations and Theses Global	noft((Eating disorder* OR Feeding and eating disorder* OR feeding NEAR/3 eating disorder* OR Appetite disorder* OR feeding disorder* OR eating and feeding disorder* OR eating NEAR/3 feeding disorder* OR restrict* NEAR/3 eat* OR anorexi* OR bulimi* OR arfid OR "Avoidant restrictive food intake disorder" OR bing* OR purg* OR "Night eating syndrome" OR Rumination NEAR/3 disorder* OR Rumination NEAR/3 syndrome OR pica OR allotriophagy OR geophag* OR "Other specified feeding and eating disorder" OR osfed OR "Unspecified feeding and eating disorder" OR ufed OR "Eating disorder not otherwise specified" OR ednos) ***AND*** (multifamil* OR mult*famil* OR mult* NEAR/2 famil* OR famil* NEAR/1 group* OR mft OR mftg OR mfg OR mfgt))
SCOPUS	TITLE-ABS-KEY ("Eating disorder*" OR "Feeding and eating disorder*" OR "feeding W/3 eating disorder*" OR "Appetite disorder*" OR "feeding disorder*" OR "eating and feeding disorder*" OR "eating W/3 feeding disorder*" OR "restrict* W/3 eat*" OR anorexi* OR bulimi* OR arfid OR "Avoidant restrictive food intake disorder" OR bing* OR purg* OR "Night eating syndrome" OR "Rumination W/3 disorder*" OR "Rumination W/3 syndrome" OR pica OR allotriophagy OR geophag* OR "Other specified feeding and eating disorder*" OR osfed OR "Unspecified feeding and eating disorder*" OR ufed OR "Eating disorder not otherwise specified*" OR ednos) ***AND*** (multifamil* OR mult*famil* OR mult* W/2 famil* OR famil* W/1 group* OR mft OR mftg OR mfg OR mfgt)
Web of Science	(TS=("Eating Disorder*") OR TS=("Feeding and eating disorder*") OR TS=("feeding W/3 eating disorder*") OR TS=("Appetite disorder*") OR TS=("feeding disorder*") OR TS=("eating and feeding disorder*") OR TS=("eating W/3 feeding disorder*") OR TS=("restrict* W/3 eat*") OR TS=(Anorexi*) OR TS=(Bulimi*) OR TS=(ARFID) OR TS=("Avoidant restrictive food intake disorder") OR TS=(Bing*) OR TS=(Purg*) OR TS=("Night eating syndrome") OR TS=("Rumination W/3 disorder*") OR TS=("Rumination W/3 syndrome") OR TS=(Pica) OR TS=(allotriophagy) OR TS=(Geophag*) OR TS=("Other specified feeding and eating disorder*") OR TS=(OSFED) OR TS=("Unspecified feeding and eating disorder*") OR TS=(UFED) OR TS=("Eating disorder not otherwise specified*") OR TS=(EDNOS)) AND (TS=(Multifamil*) OR TS=(Mult*famil*) OR TS=(Mult* W/2 famil*) OR TS=(Famil* W/1 group*) OR TS=(MFT) OR TS=(MFTG) OR TS=(MFG) OR TS=(MFGT))

NB: a separate review protocol was not prepared for this systematic scoping review
